# Implementing workplace health promotion in nursing – A process evaluation in different care settings

**DOI:** 10.1186/s12912-024-02272-6

**Published:** 2024-08-22

**Authors:** Jasmin Lützerath, Hannah Bleier, Madeleine Gernert, Andrea Schaller

**Affiliations:** 1 German Sport University Cologne, 50933 Cologne, Germany; 2 Institute for Workplace Health Promotion, 51063 Cologne, Germany; 3 Institute for Sports Science, University of the Bundeswehr Munich, 85577 Neubiberg, Germany

**Keywords:** Nursing staff, Employee health, Workplace health promotion, Occupational health and safety, Implementation and process evaluation, Logic model

## Abstract

**Objective:**

Workplace health promotion (WHP) in Germany is receiving increasing support from health insurance funds. Nevertheless, there is hardly any knowledge on the process of how health outcomes are achieved, especially in nursing. The aim of the study was to find out how and what can be implemented in different care settings and to examine the reactions and interactions of the participants under routine conditions.

**Methods:**

Guided by a logic model, a holistic WHP approach was implemented in four acute care hospitals, seven inpatient care facilities and four outpatient care services from April 2021 to October 2022. Data on realized WHP interventions, participant assessment and topics of work design was collected and analyzed descriptively.

**Results:**

The realized WHP interventions were adapted depending on the content and context. Mainly short relaxation interventions were delivered or those with an event character were received by participants. The highest participation rate of planned participants was achieved in team building training. Participants predominantly assessed WHP interventions as useful, the quality as (very) good and were generally (very) satisfied with the intervention components. For work design topics, intentions for the design of work organization were mainly documented in action plans.

**Conclusion:**

Cooperation with practitioners in research should be continued as a contribution to quality development. This could provide suggestions as to which content adjustments lead to greater acceptance by the target group in a specific context.

**Trial registration:**

The project was registered in the German Clinical Trial Register (DRKS00024961, 2021/04/09).

## Introduction

Public health is of great interest worldwide and health promotion therefore also requires efforts outside the health sector [1]. The workplace appears to be a suitable place to promote the health of employees [2–4]. Workplace health promotion (WHP) is about the combined efforts of employers, employees and society to create safe and healthy working conditions and to encourage the personal development to improve well-being and health at work [1–4]. In Germany, WHP is supported by health insurance funds [5]. The focus is in supporting the establishment and strengthening of health-promoting structures [5]. In this context, health insurers demand that WHP follows a process approach that should be addressed with company representatives (decision-makers) of all persons involved in health promotion and employee health [6]. Since the *Care Staff Strengthening Act* [7], health insurance funds in Germany have been increasingly called upon to support the health of nursing employees in particular, as this target group plays a key role in public health. Especially in care settings, it is assumed that there are different health-related needs for action due to setting-specific contexts in acute care hospitals (ACH), inpatient care facilities (ICF) and outpatient care services (OCS) [8]. To address these needs, health insurance funds are supporting a growing number of care organizations each year in establishing a WHP approach, which results in increasing costs [9, 10].

Given the increasing costs, there is a strong demand for evidence to legitimize spending on preventive and health promotion interventions [11, 12]. In Germany, previous WHP intervention studies in nursing have mainly focused on individual coping skills to promote mental or physical health, which showed no or only minor effects on the outcomes [13]. While internationally such behavioral prevention components of the WHP appear to be cost-effective for both employers and society [14]. However, it is known that a holistic WHP approach with a combination of interventions at organizational and individual level is likely to be most effective [15]. Interventions with several components that are connected or related to each other and can influence each other are also referred to as complex interventions [16]. Complexity increases further when, as in WHP, different groups or organizational levels in the work environment are addressed and the process approach increases flexibility in terms of the content of interventions [13, 14]. As a result, the evaluation of complex interventions also becomes complex, as the interaction between content and context determines and shapes whether and how outcomes are achieved [17, 18]. Therefore, a complementary process evaluation can provide important insights associated with variation in outcomes [17].

Developing evidence requires a combination of scientific evidence and the practical experience of relevant professionals, such as WHP experts, as well as the perspective of the target population [11]. Still, practitioners tend to see evaluation as an additional task and therefore data that can be used to adjust programs is rarely collected [19]. Specifically, concurrent process evaluations alongside effectiveness studies for WHP programs are hardly conducted, and the quality of studies in this regard could be improved [20].

It appears that there is a lack of systematic and high quality research on WHP [21–23], and evidence is especially limited in the care sector [13, 24, 25]. In this context, neither the feasibility nor the impact of WHP interventions can be interpreted without a theoretical basis [26]. A possible solution to address the complexity of evidence development could be to work with a theory of change, such as in the form of a logic model that can represent a results chain [27]. Thereby, logic models emphasize the relevance of process evaluation alongside outcome evaluation, taking into account the context [28]. Moreover, by graphically representing the assumed causal linkages, a logic model can provide a communication basis for collaboration with all stakeholders involved in WHP [29, 30]. This could be beneficial as it is useful for evaluation purposes to agree with practitioners which data can be collected under routine conditions [28, 31]. Some WHP interventions have already been systematically evaluated guided by a logic model [32–35].

The study presented was part of a model project, which aimed to contribute to the promotion of health and improvement of the working situation of nursing employees in different care settings. The conception and evaluation of the implemented WHP approach was based on a program logic model. The process evaluation was conducted with the objectives of examining the implementation of a holistic WHP approach and the participants’ response to and interaction with the interventions under routine conditions in different nursing settings. Based on the practicability of data collection, this led to the following research questions:

1. Which WHP interventions were realized in different care settings?
2. How do the participants assess the WHP interventions in the care organizations?
3. Which topics of work design were addressed by WHP?

## Materials and methods

The process evaluation was conducted as part of the project “Workplace offers for health promotion and violence prevention” (BAGGer), funded by the German Federal Ministry of Health. The study was registered in the German Register for Clinical Studies (DRKS-ID: DRKS00024961) and approved by the Ethics Committee of the German Sport University Cologne (No. 097/2021; 07 June 2021).

### Logic model guiding the project

As a guideline for the conception and evaluation of the implemented WHP approach, a logic model was created in cooperation with scientists and practitioners, which was filled with information from the target population [36].

Concerning the planned work, theoretical assumptions for intervention reasoning (Assumptions) were obtained from the literature in the form of systematic reviews [13, 37]. Afterwards, different analysis methods were used to describe with which means and resources (Inputs) were needed. For this purpose, qualitative interviews were conducted with nurses and complemented by a quantitative employee survey in order to identify relevant health burdens of nursing care employees and determine promising interventions [38, 39]. In addition, structures for the participatory design of a WHP approach were created involving the target group, employers and WHP experts. Based on the Inputs, the WHP interventions (Activities) were carried out in each care setting under routine conditions at an organizational and individual level according to the German “Guideline Prevention” (“Leitfaden Prävention”) [6].

The intended results have been assessed in terms of direct products of the interventions in the process (Outputs). Based on this, future publications of the project could reveal changes in perceived work demands and health behavior at work (Outcomes) in order to determine whether WHP could contribute to fundamental changes (Impact).

Possible Context Factors, which include organizational framework conditions for workplace health management and facilitators and barriers to participation in WHP, were identified in the form of interviews with managers and employees [40, 41].

**Fig. 1:**
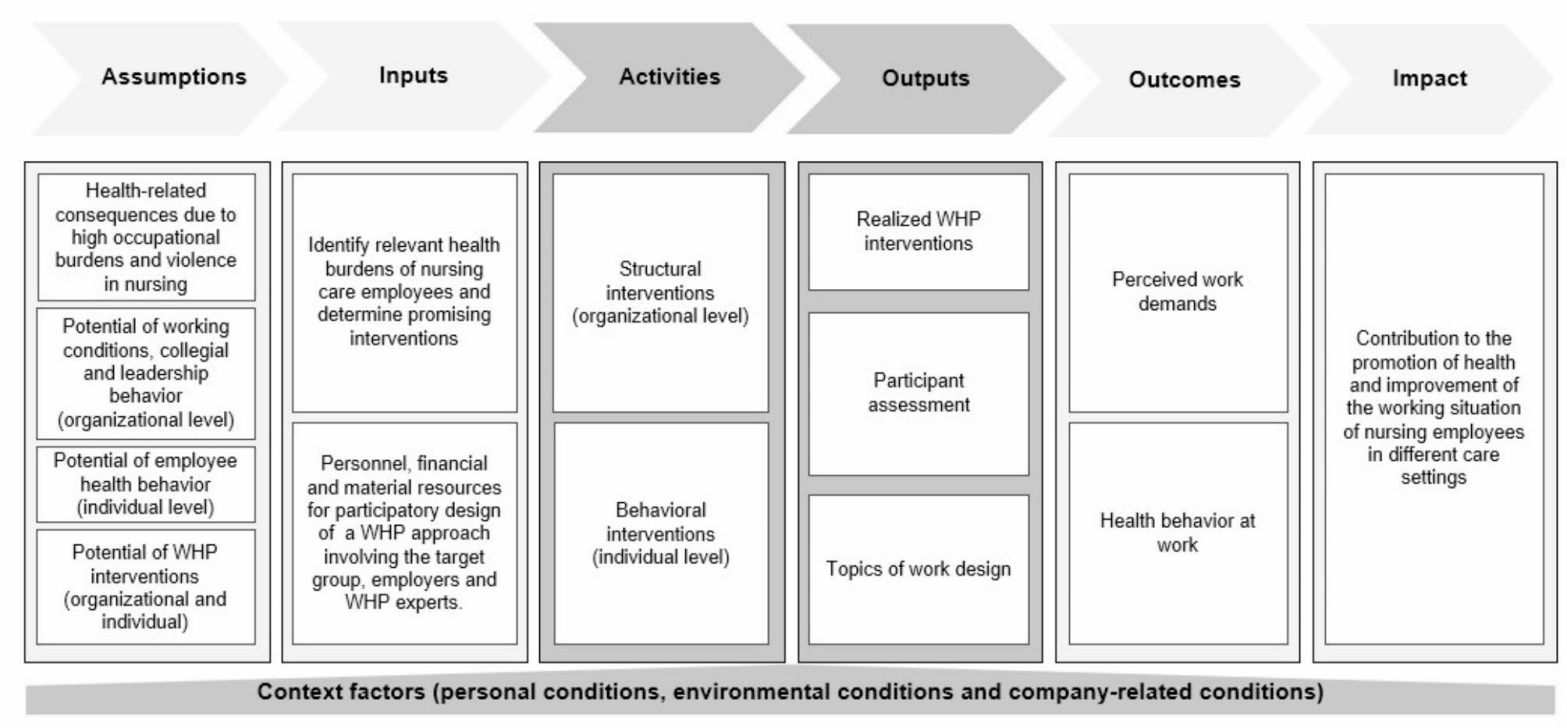
Logic model guiding the project

An illustration of the logic model guiding the project can be found in Fig. 1.

### Participants and setting

The systematic implementation of the WHP approach took place between April 2021 and October 2022 in 15 care organizations (ACH = 4, ICF = 7, OCS = 4) in North Rhine-Westphalia, Germany. All 3312 employees (ACH = 2127, ICF = 506, OCS = 679) who were working in one of the care organizations during the project period were able to participate in the interventions. Participation was voluntary and individual participants were recruited by the decision-makers of the organizations.

### The holistic WHP approach

The interventions were carried out by WHP experts, all of whom had experience in care settings, under routine conditions on behalf of a health insurance company. In order to establish and strengthen health-promoting structures, a Kick Off meeting was held in each care organization to link a steering committee to existing structures and processes. Interventions were planned for each care organization in a participatory design by WHP experts and decision-makers. The content was largely driven by company-specific needs for action and was based on existing interventions by WHP experts on an organizational and individual level. *Structural interventions* for health-promoting work design address organizational topics of work environment, task design, work organization and social relations [6]. *Behavioral interventions* to promote a healthy work and lifestyle address individual coping skills and include the topics of stress management and strengthening resources, physical-activity-promoting work and physically active employees, healthy nutrition in everyday working life, and addiction prevention [6]. The content could be delivered in the form of meetings, consultations, workshops, trainings, sessions and event days, with recommendations for the duration of the units and the number of participants.

Adaptation of the recommendations was made on a company-specific basis depending on available resources. The personnel resources for attending the interventions were provided by the participating care organizations. All interventions were performed during working hours or could be credited as working hours. The intervention costs were fully and indefinitely covered by the health insurance company during the project. Meeting rooms of the individual care organizations were used as places of implementation.

### Study design

This process evaluation was based on the Medical Research Council guidance for conducting and reporting process evaluation [28]. The key components of a process evaluation interact with the context and include, the *implementation*, the what and the how, as well as the *mechanisms of impact* that show how the delivered interventions produce change [28].

In terms of *implementation*, fidelity was defined as whether the WHP interventions were realized in accordance with the recommendations of the WHP experts. The dose refers to the amount of interventions delivered in each setting and received by participants. Reach was defined as whether the planned participants were attending.

Regarding the *mechanism of impact*, two pathways in which participants respond to and interact with the interventions were considered. Firstly, participants could assess the interventions directly, and secondly, employees could be influenced indirectly through the changes in work design.

### Data collection and analysis

In terms of the process evaluation, multicenter data collection was conducted concurrently with implementations to formatively evaluate the holistic WHP approach post-process. The WHP experts provided a description, including the recommendations, of the implemented interventions and categorized them according to the WHP fields of action and the intended need for action. In order to answer the research questions, data on realized WHP interventions, participant assessment and topics of work design was collected by the accompanying WHP experts (see Table 1).

The WHP interventions were documented before and after being realized. The content as well as the number of implementations, the duration and the number of planned participants were determined in advance in consultation with the decision-makers of the care organizations. The actual number of attending participants was documented on the day of implementation. In order to obtain a participant assessment, a questionnaire was distributed and collected after trainings or sessions. Topics of work design emerged during meetings, consultations or workshops and were documented by WHP experts in company-specific action plans. The intentions in the action plans were continuously planned, implemented, and subsequently reviewed by the decision-makers in the steering committee under the moderation of the WHP experts according to need and urgency. At the end of the project, the status of implementation was determined for all intentions.

The data was analyzed descriptively (frequencies, means and standard deviations).

**Table 1:** Operationalization of output

Output	Operationalization	Response Option
Realized WHP interventions	Dose delivered	Number of implementations
	Duration	Intervention unit time
	Reach	Participation rate: “Number of participants attended”/ “Planned number of participants”
Participant assessment	Usefulness (fulfillment of expectations, content-related benefit*, learning experience*, motivation to practice the content)	4-point scale (agree – disagree)
	Structural quality [42] (announcement*, rooms, media, group composition)	5-point scale (very good – very poor)
	Process quality [42] (comprehensibility*, communication of content*, responsiveness to questions, relevance to everyday life*)	5-point scale (very good – very poor)
	Satisfaction (with the training in general*)	4-point scale (very satisfied – dissatisfied)
Topics of work design	Work environment [6] (physical factors, ergonomics, work equipment, operational conditions)	Number of intentions in the action plans
	Task design [6] (decision latitude, qualification, holistic nature of the task, physical requirements)	Number of intentions in the action plans
	Work organization [6] (information, working hours, workflow, cooperation)	Number of intentions in the action plans
	Social relations [6] (leadership, colleagues, customers, clients)	Number of intentions in the action plans
	Implementation status (completed, in progress, planned)	Number of implemented intentions at the end of the project

^*^Included in the abbreviated questionnaire for interventions with a duration of < 60 min.

## Results

Interventions were classified by WHP experts in the fields of action according to the need for action. A need for action was seen in shaping the demands of everyday work, in coping with social, mental or physical burdens and creating awareness of WHP interventions. A classification and description of the interventions delivered, including the WHP experts’ recommendations on the duration and number of participants per implementation, can be found in Table 2.

**Table 2:** Description of the interventions

Name	Description	Duration	Number of participants
***Structural interventions***			
**Shaping the demands of everyday work**			
*Kick Off*	At an initial meeting with representatives of all persons involved in health promotion and occupational health and safety (establishment or expansion of the steering committee), a common understanding of health promotion is defined, and goals are set.	3–4 h	At least 5 decision makers
*Steering committee*	Quarterly workgroup meeting to determine needs, plan and implement WHP offerings and topics, communicate and evaluate health promotion process.	1.5–3 h	At least 5 decision makers
*Employee workshop * *on work design*	In moderated group discussions, employees analyze their own work situation. Concrete solution ideas are developed for identified problems and presented to the decision-makers to develop an action plan.	2–4 h	8–12 employees
*Ergonomics consulting*	On-site guidance of the work process and creation of written recommendations for a work environment conducive to physical activity.	One to several days	At least one dedicated person per department
*Movement scout*	Multiplier workshop to develop skills to offer small movement sessions to colleagues.	3–5 × 2 h	Maximum 10 Participants
**Coping with social burdens**			
*Leadership training*	Training on the topics: Self-care as a role model function as a leading employee and health-oriented employee management.	4–8 h	At least 4 leading employees
*Team building training*	Group workshop with the focus on social interaction with each other and development respectful cooperation in teams.	3–6 h	8–12 employees
*Communication training*	Training on expectations and attitudes in conversations as well as de-escalating conversation techniques.	6–8 h	8–12 employees
*Violence prevention training*	Training in violence prevention and de-escalation, recognizing situations that trigger violence and dealing with aggressive behavior	6–8 h	8–12 employees
***Behavioral interventions***			
**Coping with mental burdens**			
*Stress management training*	Behavioral training for stress management and to strengthening of psychological resources.	4–8 h	8–12 employees
*Relaxation session*	Practicing relaxation and regeneration techniques that can be integrated into the daily work routine or during breaks.	20–60 min	1–5 employees
**Coping with physical burdens**			
*Back health training*	Training on basic tension in the body, relieving postures in care, and body awareness and muscle building exercises.	2 × 3–4 h	8–12 employees
*Movement session*	Practicing movement techniques for relieve back and neck strain that can be integrated into the daily work routine or during breaks.	20–60 min	1–5 employees
**Creating awareness of WHP offers**			
*Health promotion * *event day*	Event day to promote WHP activities face-to-face with the aim of making employees aware of their health and health prevention and motivate them to adopt a healthy work and lifestyle.	3–8 h	At least 20 employees

h = hours

### Realized WHP interventions

The realized WHP interventions in the 15 care organizations participating in the project is shown in comparison of the settings (see Table 3).

Regarding fidelity, adaptations were made in all settings to the *steering committee* with regard to the recommended duration; the meetings were shorter. Furthermore, only one *movement scout* unit was implemented in ACH instead of the recommended three, and the duration of the *leadership training* and *movement sessions* was also shortened through adaptations. In all settings, the number of planned participants in *team building training* was increased beyond the recommendation, which was also adapted for *stress management training* in OCS. In contrast, fewer participants than recommended were planned for *employee workshop on work design* in ICF and OCS and for *communication training* and *violence prevention training* in OCS.

In all settings, a higher dose was delivered in *behavioral interventions* than in the *structural interventions*. The highest dose delivered in all settings was achieved in the intervention *relaxation session* with a total of 185 implementations (ACH = 27, ICF = 112, OCS = 45). For dose received by participants, most had contact with the *health promotion event days* across all settings, with 474 participants attending (ACH = 323, ICF = 65, OCS = 86). Whereas in ICF, the highest dose received by participants was in the intervention component *relaxation session* with 212 attending participants.

In terms of reach, a total of 1680 participants attended the structural and behavioral interventions (ACH = 757, ICF = 608, OCS = 315), which corresponds to a participation rate of 77.9% (ACH = 78.2%, ICF = 86.7%, OCS = 64.5%).

**Table 3:** Realized WHP interventions

	All care settings *n* = 15 3312 employees	Acute care hospitals *n* = 4 2127 employees	Inpatient care facilities *n* = 7 506 employees	Outpatient care services *n* = 4 679 employees
***Structural interventions (total n)***	***137***	***52***	***53***	***32***
**Shaping the demands of everyday work**				
*Kick Off* Dose delivered [n] Duration min/n [min-max] Reach [att./plan. (%)]	15 180–240 100/n.a.	4 180–240 45/n.a.	7 180–240 33/ n.a.	4 180–240 22/n.a.
*Steering committee* Dose delivered [n] Duration min/n [min-max] Reach [att./plan. (%)]	65 30 – 180 303/438 (69.2%)	21 30–120 123/196 (62.8%)	28 60 – 180 120/158 (75.9%)	16 45 – 150 60/84 (71.4%)
*Employee workshop on * *work design* Dose delivered [n] Duration min/n [min-max] Reach [att./plan. (%)]	26 180 – 240 184/222 (82.9%)	15 180–240 110/143 (76.9%)	4 240 29/29 (100%)	7 180–240 45 /50 (90.0%)
*Ergonomics consulting* Dose delivered [n] Duration min/n [min-max] Reach [att./plan. (%)]*	3 360 18/18 (100%)	0 - -	3 360 18/18 (100%)	0 - -
*Movement scout* Dose delivered [n] Duration min/n [min-max] Reach [att./plan. (%)]	1 120 8/8 (100%)	1 120 8/8 (100%)	0 - -	0 - -
**Coping with social burdens**				
*Leadership training* Dose delivered [n] Duration min/n [min-max] Reach [att./plan. (%)]	10 180 – 390 85/112 (75.9%)	8 180–390 68/92 (73.9%)	2 240 17/20 (85.0%)	0 - -
*Team building training* Dose delivered [n] Duration min/n [min-max] Reach [att./plan. (%)]	5 240 – 360 68/77 (88.3%)	2 360 29/32 (90.6%)	2 240 24/30 (80.0%)	1 360 15/15 (100%)
*Communication training* Dose delivered [n] Duration min/n [min-max] Reach [att./plan. (%)]	6 360 45/50 (90.0%)	0 - -	3 360 28/28 (100%)	3 360 17/22 (77.3%)
*Violence prevention training* Dose delivered [n] Duration min/n [min-max] Reach [att./plan. (%)]	6 360–390 55/67 (82.1%)	1 390 10/12 (83.3%)	4 360–390 43/52 (82.7%)	1 390 2/3 (66.7%)
***Behavioral interventions (total n)***	***235***	***69***	***117***	***49***
**Coping with mental burdens**				
*Stress management training* Dose delivered [n] Duration min/n [min-max] Reach [att./plan. (%)]	1 240 12/14 (85.7%)	0 - -	0 - -	1 240 12/14 (85.7%)
*Relaxation session* Dose delivered [n] Duration min/n [min-max] Reach [att./plan. (%)]	185 20–45 296/374 (79.1%)	27 20 23/36 (63.9%)	112 20–45 217/ 250 (86.8%)	46 20–30 56/88 (63.6%)
**Coping with physical burdens**				
*Back health training* Dose delivered [n] Duration min/n [min-max] Reach [att./plan. (%)]	2 240 14/19 (73.7%)	0 - -	2 240 14/19 (73.7%)	0 - -
*Movement session* Dose delivered [n] Duration min/n [min-max] Reach [att./plan. (%)]	38 15 18/38 (47.4%)	38 15 18/38 (47.4%)	0 - -	0 - -
**Creating awareness of WHP interventions**				
*Health promotion event day* Dose delivered [n] Duration min/n [min-max] Reach [att./plan. (%)]	9 270–540 474/720 (65.8%)	4 390–540 323/411 (78.6%)	3 390 65/ 97 (67.0%)	2 270–390 86/212 (40.6%)

Dose delivered = Number of implementations; n = number; Reach = Participation rate; att. = number of participants attended; plan. = planned number of participants; n.a. = not available; * = departments

### Participant assessment

In all settings, out of a total of 593 participants who attended trainings and sessions, 432 completed the questionnaire, which corresponds to a response rate of 72.8%. The response rate was highest among the 102 OCS participants (86.3%), followed by the 343 ICF participants (83.7%), and lowest among the 148 ACH participants (38.5%).

Usefulness was rated from 1.1 (± 0.3) to 2.0 (± 0.8) on a 4-point scale. Structural quality from 1.1 (± 0.2) to 2.1 (± 1.0) and process quality from 1.0 (± 0.0) to 1.6 (± 0.7), each on a 5-point scale. Satisfaction was rated on a 4-point scale from 1.0 (± 0.0) to 2.0 (± 0.8).

A detailed participant assessment of the different trainings can be found in Table 4.

**Table 4:** Participant assessment of trainings

	All care settings *n* = 15 3312 employees	Acute care hospitals *n* = 4 2127 employees	Inpatient care facilities *n* = 7 506 employees	Outpatient care services *n* = 4 679 employees
***Structural interventions***				
Coping with ***social burdens***				
*Leadership training [n/att. (%)]* Usefulness [M (± SD)] * Structural quality [M (± SD)] ** Process quality [M (± SD)] ** Satisfaction [M (± SD)] ***	30/85 (35.3%) 1.4 (± 0.6) 1.3 (± 0.5) 1.2 (± 0.4) 1.2 (± 0.4)	13/68 (19.1%) 1.7 (± 0.6) 1.5 (± 0.6) 1.4 (± 0.5) 1.5 (± 0.5)	17/17 (100%) 1.2 (± 0.4) 1.2 (± 0.4) 1.1 (± 0.3) 1.0 (± 0.0)	- - - - -
*Team building training [n/att. (%)]* Usefulness [M (± SD)] * Structural quality [M (± SD)] ** Process quality [M (± SD)] ** Satisfaction [M (± SD)] ***	29/68 (42.6%) 1.3 (± 0.5) 1.5 (± 0.7) 1.2 (± 0.4) 1.2 (± 0.4)	6/29 (20.7%) 1.3 (± 0.5) 1.5 (± 0.6) 1.1 (± 0.3) 1.2 (± 0.4)	8/24 (33.3%) 1.2 (± 0.4) 1.5 (± 0.6) 1.3 (± 0.4) 1.1 (± 0.4)	15/15 (100%) 1.3 (± 0.5) 1.4 (± 0.7) 1.1 (± 0.3) 1.2 (± 0.4)
*Communication training [n/att. (%)]* Usefulness [M (± SD)] * Structural quality [M (± SD)] ** Process quality [M (± SD)] ** Satisfaction [M (± SD)] ***	45/45 (100%) 1.3 (± 0.6) 1.5 (± 0.6) 1.2 (± 0.5) 1,3 (± 0.5)	- - - - -	28/28 (100%) 1.4 (± 0.7) 1.6 (± 0.7) 1.3 (± 0.5) 1.4 (± 0.5)	17/17 (100%) 1.1 (± 0.4) 1.4 (± 0.6) 1.1 (± 0.3) 1.1 (± 0.2)
*Violence prevention training [n/att. (%)]* Usefulness [M (± SD)] * Structural quality [M (± SD)] ** Process quality [M (± SD)] ** Satisfaction [M (± SD)] ***	37/55 (67.2%) 1.5 (± 0.7) 1.7 (± 0.8) 1.4 (± 0.6) 1.5 (± 0.7)	9/10 (90.0%) 2.0 (± 0.8) 2.1 (± 1.0) 1.6 (± 0.7) 2.0 (± 0.8)	28/43 (65.1%) 1.3 (± 0.6) 1.6 (± 0.6) 1.4 (± 0.5) 1.4 (± 0.6)	0/2 (0.0%) - - - -
***Behavioral interventions***				
Coping with ***mental burdens***				
*Stress management training [n/att. (%)]* Usefulness [M (± SD)] * Structural quality [M (± SD)] ** Process quality [M (± SD)] ** Satisfaction [M (± SD)] ***	12/12 (100%) 1.1 (± 0.3) 1.3 (± 0.5) 1.0 (± 0.0) 1.1 (± 0.3)	- - - - -	- - - - -	12/12 (100%) 1.1 (± 0.3) 1.3 (± 0.5) 1.0 (± 0.0) 1.1 (± 0.3)
*Relaxation session [n/att. (%)]* Usefulness [M (± SD)] * Structural quality [M (± SD)] ** Process quality [M (± SD)] ** Satisfaction [M (± SD)] ***	254/296 (85.8%) 1.2 (± 0.5) 1.2 (± 0.5) 1.1 (± 0.3) 1.1 (± 0.3)	11/23 (47.8%) 1.4 (± 0.7) 1.3 (± 0.5) 1.2 (± 0.3) 1.1 (± 0.3)	199/217 (91.7%) 1.2 (± 0.5) 1.2 (± 0.4) 1.1 (± 0.3) 1.1 (± 0.3)	44/56 (78.6%) 1.2 (± 0.4) 1.4 (± 0.8) 1.1 (± 0.3) 1.1 (± 0.3)
Coping with ***physical burdens***				
*Back health training [n/att. (%)]* Usefulness [M (± SD)] * Structural quality [M (± SD)] ** Process quality [M (± SD)] ** Satisfaction [M (± SD)] ***	7/14 (50.0%) 1,3 (± 0,7) 1,4 (± 0,6) 1,0 (± 0,2) 1,2 (± 0,4)	- - - - -	7/14 (50.0%) 1.3 (± 0,7) 1.4 (± 0,6) 1.0 (± 0,2) 1.2 (± 0,4)	- - - - -
*Movement session [n/att. (%)]* Usefulness [M (± SD)] * Structural quality [M (± SD)] ** Process quality [M (± SD)] ** Satisfaction [M (± SD)] ***	18/ 18 (100%) 1.1 (± 0,3) 1.1 (± 0,2) 1.1 (± 0,3) 1.2 (± 0,4)	18/ 18 (100%) 1.1 (± 0,3) 1.1 (± 0,2) 1.1 (± 0,3) 1.2 (± 0,4)	- - - - -	- - - - -

n = number of completed feedback forms; att. = number of participants attended; *= [1 (agree) – 4 (disagree)]; **= [1 (very good) – 5 (very poor)]; ***= [1(very satisfied) – 4 (dissatisfied)]

### Topics of work design

Based on the topics of work design emerged during meetings, consultations or workshops, a total of 121 (ACH = 60; ICF = 32; OCS = 29) intentions were made by decision-makers in the steering committee and documented in action plans. In all care settings, it was primarily topics relating to *work organization* (53.7%) that were discussed in detail and addressed in a solution-oriented manner. In detail, it was the “workflow” in ACH and ICF, while in OCS it was mainly the passing on of “information”. At the end of the project 62.8% of the intentions were implemented and 25.6% in progress. Further detail can be found in Table 5.

**Table 5:** Topics of work design according to the “Guideline Prevention”

	All care settings *n* = 15 3312 employees	Acute care hospitals *n* = 4 2127 employees	Inpatient care facilities *n* = 7 506 employees	Outpatient care services *n* = 4 679 employees
***Number of intentions in the action plans (total n)***	***121***	***60***	***32***	***29***
*Work environment [n (%)]* Physical factors [n] Ergonomics [n] Work equipment [n] Operational conditions [n]	32 (26.4%) 2 8 7 15	17 (28.3%) 2 1 4 10	11 (34.4%) - 5 2 4	4 (13.8%) - 2 1 1
*Task design* Decision latitude [n] Qualification [n] Holistic nature of the task [n] Physical requirements [n]	14 (11.6%) 3 7 4 -	6 (10.0%) 1 3 2 -	2 (6.3%) - 1 1 -	6 (20.7%) 2 3 1 -
*Work organization* Information [n] Working hours [n] Workflow [n] Cooperation [n]	65 (53.7%) 23 9 27 6	34 (56.7%) 10 5 16 3	15 (46.8%) 5 2 6 2	16 (55.2%) 8 2 5 1
*Social relations* Leadership [n] Colleagues [n] Customers [n] Clients [n]	10 (8.3%) 6 4 - -	3 (5.0%) 1 2 - -	4 (12.5%) 2 2 - -	3 (10.3%) 3 - - -
Implementation status* *[n]* Completed [n; %] In progress [n; %] Planned [n; %]	121 76 (62.8%) 31 (25.6%) 14 (11.6%)	60 33 (55.0%) 16 (26.7%) 11 (18.3%)	32 21 (65.6%) 10 (31.3%) 1 (3.1%)	29 22 (75.9%) 5 (17.2%) 2 (6.9%)

n = number; % = proportion of intentions in relation to all intentions of the action plans per setting; * = at project end

## Discussion

The aim of this process evaluation was to examine the implementation of a holistic WHP approach at both organizational and individual level, and the response of participants to and interaction with it under routine conditions in different care contexts. A key finding was that implementation is shaped by content and context. In realized WHP interventions, there were adjustments during implementation that differed from the recommendations of the WHP experts (fidelity). With regard to fidelity, the duration was shortened, particularly in the ACH context when the content was familiar, while the planned number of participants was reduced in the contexts with higher sick leave (ICF, OCS). Regardless of the context, the number of participants in the content *team building training* and *stress management training* was increased. In the case of the amount of realized WHP interventions (dose), *relaxation sessions* were mainly delivered and these short WHP interventions or *health promotion event day*s were received by participants, whereby both serve more to raise awareness of health topics than to bring about a long-term change in behavior. The highest participation rates (reach) in realized WHP interventions were achieved in the ICF context and in the content of *team building training*. In terms of participant assessment, the interventions were predominantly rated as useful and (very) good/satisfied by participants when they were well announced. Furthermore, the number of intentions to address topics of work design seems to increase with the number of *employee workshops on work design* delivered. Employee participation appears to be the key to meeting the need for action on *work organization* topics and achieving a high level of implementation.

### Realized WHP interventions

Adaptions to the realized WHP interventions were made depending on the resources available in the care organizations, whereby a closer look at the fidelity revealed a far more differentiated picture. A *steering committee* was linked to existing structures and processes in all organizations, which resulted in a shorter duration in all settings by adapting to a company-specific context. At the start of the project, all ACHs were known to already have a *steering committee*, compared with 71% of ICFs and 60% of OCSs [40]. The existence of comparable content could be a reason why fewer time resources are provided for planning. This is also reflected in the fact that in ACH the *movement scout* intervention was delivered less frequently than recommended and *leadership training* and *movement sessions* were delivered for a shorter period. In Germany, 94% of ACHs already offer leadership training and movement courses are available in 90% of them [43]. Further changes were made to the interventions with regard to the human resources made available. *Employee workshops on work design* were planned in ICF and OCS with fewer participants than recommended. Here the reason could be the context, since during the implementation phase sick leave was higher in ICF than OCS and lowest in ACH [44, 45]. In contrast, more participants were planned for *team building training* than recommended in all settings and for *stress management training* in OCS. This greater interest could be due to the fact that the perceived social work climate is one of the key influencing factors on employee health in nursing [39]. Stress management, good communication and social support are particularly important for OCS nurses in Germany [46, 47]. Therefore, it is surprising that *communication* or *violence prevention trainings* in OCS were planned with fewer participants than recommended. This could be due to the context, as OCS was the setting with the lowest number of (verbal) violence incidents at the start of the project [39].

This study showed that the dose delivered of behavioral interventions was higher than of structural interventions. This tendency in the thematic scope of the dose delivered is also reflected in the data from the reporting system of the health insurance funds [9, 10]. The fit of WHP interventions with shift times and participating during work time seem to be crucial in planning [41], which could be one reason why mainly short *relaxation sessions* were delivered. In addition to the care setting context, the content could also be decisive for delivery. Contrary to expectations, *stress management training* was only implemented once in OCS, although both ACH and OCS employees showed high levels of stress indicators at the start of the project [39]. A preference for relaxation content over stress management interventions was also seen in other studies, despite lower effects on psychological outcome variables [48]. Besides, a great need for action is seen in Germany for the nursing sector on the topics of back health and strengthening [47]. In the care setting comparison, it is noticeable that *ergonomics consulting*,* back health trainings* and short *movement sessions* were only delivered in ACH and ICF, although it is known that physical workload is particularly demanding in both ICF and OCS [38, 49]. One reason could be that OCS work takes place in the context of private households, which could complicate the implementation of *ergonomics consulting*, although a lot of time is also spent in the car, where adaptation to physiological needs could be useful [38, 49]. Emotional exhaustion is perceived much more frequently than physical complaints in OCS contexts [50], so it may be that this is a higher priority in the planning process. While the ICF participants received the highest dose in the *relaxation sessions*, the highest dose in ACH and OCS was received in *health promotion event day*s. In this context, *health promotion event day*s that provide a space for face-to-face outreach can be a useful complement to communication, as they create opportunities for personal contact [51]. Especially nurses want direct face-to-face communication when it comes to WHP in addition to written communication via email or notice boards [41].

The reach of the holistic WHP approach in this study was a participation rate of 77.9% in all settings. It is well known that participation in WHP is often below 50% [52]. The results also showed predominantly higher participation rates per intervention, which were between 40.6% and 100% in all care settings. Comparable participation rates (38.5–100%) were also seen in other holistic WHP approaches in nursing [53]. In a comparison of settings, the highest participation rate was achieved in ICF and the lowest in OCS. It is known that, particularly in nursing, a high work density tends to inhibit participation, while support from management tends to encourage participation [41, 54, 55]. Supporting this, the perceived quantitative workload was lowest in the ICF context [39]. The health-promoting willingness of managers at the start of the project, on the other hand, was highest in the OCS context [40]. However, in OCS it is also known that the distance between the place of living and the company as the place of implementation can also inhibit participation [41, 56], which in turn could be another reason for the low level of participation. *Team building training* was an intervention that was delivered in all settings and had the highest overall participation rate of 88.3% over a six-hour period. It is known that the expectation of health benefits from the content can facilitate participation [57, 58]. In contrast to the dose, this is confirmed by the fact that the highest participation rates (100%) were found for interventions with a longer duration.

### Participant assessment

The questionnaires to assess the interventions were completed by 72.8% of the participants. Participant assessment was rated useful and (very) good/ satisfied in most interventions, with the exception of *violence prevention* in ACH. However, as only nine participants took part in one training session, it remains unclear whether this is a one-off lower rating or whether the content should generally be changed for the ACH context. Other authors also found that WHP interventions tend to get high usefulness and satisfaction ratings [53, 59]. In contrast, one study that found more dissatisfaction with WHP interventions attributed this to the study design, in which participants were not informed about the content or goal of the WHP intervention [60]. In this study, the structural quality, which includes the announcement, was rated as (very) good by the participants. In this regard, it is noticeable that this one *violence prevention training* with the lowest approval ratings for usefulness and satisfaction also received the lowest rating for structural quality. Accordingly, the announcement as well as the group composition or target group could be another aspect that could be given greater consideration during WHP planning.

### Topics of work design

When it comes to changes in work design, most intentions were documented in the ACH action plans (60 in 4 organizations) and the fewest in the ICF (32 in 7 organizations). This is also reflected in the number of *employee workshops on work design* in the settings that were conducted most in ACH and least in ICF. In all setting contexts, the content was primarily intended to address *work organization* topics. In care settings, we know that work organization can be one of the main factors influencing health [39]. Therefore, it seems that the need for action has been met. On the other hand, this study was carried out in the middle of the pandemic in Germany. During this time, the dynamic infection situation meant that work organization in particular had to be rethought even without WHP consulting [61, 62]. It is noticeable that in ACH and ICF mainly workflow topics were intended, while in OCS the focus was on information topics. This could be due to the fact that in ACH and ICF in particular, work-flows have to be coordinated on site, whereas in OCS the work tends to be done alone and therefore information transfer could have a higher importance in *work organization* [38]. It is striking that there were hardly any topics on *social relations* in all settings and none with customers and clients. While some topics have already been covered through *leadership*,* team building*,* violence prevention* and *communication trainings*, this is not entirely the case with interaction work with customers and clients. This could be due to the fact that nurses assume that other work demands, such as staffing, have a considerable effect on interaction work and that these therefore take priority [38, 63]. At the end of the project, 88.4% (62.8% completed and 25.6% in progress) of all intentions in the action plans had been implemented. Other studies reported 45–86% of intentions implemented within 6 to 12 months [64]. In Nursing, perceived implementation ranges from 26 to 79% [60]. The high implementation status in this study could be related to the involvement of employees in creating them. We know that employee involvement can help to optimally adapt the intentions to the organizational culture and context, that employees feel more responsible, and that the change process runs more smoothly [65, 66].

### Strengths and limitation

A major strength of this study is that it is based on scientific evidence and a participatory design of a holistic WHP approach at organizational and individual level involving the target group of nursing employees, employers, and WHP experts. Thus, an evaluation method could be tested in practice under routine conditions.

However, the experimental design has also led to some limitations. Recruitment was carried out by decision-makers from the care organizations. In Germany, WHP interventions are thought to be more likely to be adopted by healthy and active care employees, whereas older employees who already have work-related harm should be targeted more [67, 68]. In contrast, other studies found few statistically significant associations with demographic, health, and work-related characteristics and participation besides gender [52]. Since no personal characteristics of the participants were collected, it remains unclear whether the desired target groups were reached. While in the intervention *steering committee* a repeated participation of the same persons was aimed at, in other interventions it is unclear whether persons participated more than once. The interventions were planned based on the organizations’ need for action, but it cannot be ruled out that certain content was already known from other offers. During implementation, adjustments were made depending on the available resources (context) of the care organizations and the costs of the interventions were fully covered by a health insurance company but not determined. In addition to a survey of existing content, a supplementary evaluation of context factors and economic aspects could provide interesting insights for future studies. Further, no validated/ established instruments were used to evaluate and assess the interventions. Neither were reasons for non-participation or correlations assessed and some participant assessment data was not available or missing. It is therefore uncertain whether the other participants would have assessed the different interventions better or worse. The implementation of work design topics has shown that the intentions meet the need for action. However, it remains to be determined whether these will also bring about long-term changes since no impact can be derived from descriptive data.

## Conclusion

The results presented underlined that both content and context should be taken into account when adapting WHP interventions. For practitioners, this could mean that the focus of implementation in care organizations could be more on well announced participatory and team-building content and on relaxation rather than stress management. When announcing WHP interventions in nursing, additional face-to-face contact could be an enriching element of communication. Moreover, for the development of interventions, it could be interesting to observe whether care organizations tend to accept shorter WHP interventions or those with an event character, regardless of the topic. The company-specific context, such as shift times or travel times of employees to the event location, could be given greater consideration in the planning process in order to potentially increase participation. The comparison of the care settings could also provide indications for a re-examine of some interventions. In the ACH context, this could mean that further participant assessments of violence prevention training are reviewed more intensively in order to adapt the content if necessary. Especially in the ICF context, the implementation of employee workshops on work design could be encouraged, as these seem to cover the need for action and thus contribute to creating healthy working conditions. Consideration could also be given to whether the existing content on back health and strengthening can be implemented in the OCS context.

For research, the concept of working with practitioners on a project could make a tremendous contribution to evidence and quality development in WHP. Further research could help to make the benefits of collaborative work with a logic model even clearer. To this end, further development of the logic model on outcomes and in long-term studies on impacts could be useful in the future. This could provide crucial information on what to focus on during implementation when it comes to promoting the health of nursing employees.

## Data Availability

The data presented in this study are available on request from the corresponding author.
